# HuPSON: the human physiology simulation ontology

**DOI:** 10.1186/2041-1480-4-35

**Published:** 2013-11-22

**Authors:** Michaela Gündel, Erfan Younesi, Ashutosh Malhotra, Jiali Wang, Hui Li, Bijun Zhang, Bernard de Bono, Heinz-Theodor Mevissen, Martin Hofmann-Apitius

**Affiliations:** 1 Fraunhofer Institute for Algorithms and Scientific Computing (SCAI), Schloss Birlinghoven, Sankt Augustin, Germany; 2 Bonn-Aachen International Center for Information Technology (B-IT), University of Bonn, Bonn, Germany; 3 University College London (UCI), Gower Street, WC1E 6BT, London, UK

**Keywords:** Simulation, Algorithm, Interoperability, Ontology, Semantics, Text mining

## Abstract

**Background:**

Large biomedical simulation initiatives, such as the Virtual Physiological Human (VPH), are substantially dependent on controlled vocabularies to facilitate the exchange of information, of data and of models. Hindering these initiatives is a lack of a comprehensive ontology that covers the essential concepts of the simulation domain.

**Results:**

We propose a first version of a newly constructed ontology, HuPSON, as a basis for shared semantics and interoperability of simulations, of models, of algorithms and of other resources in this domain. The ontology is based on the Basic Formal Ontology, and adheres to the MIREOT principles; the constructed ontology has been evaluated via structural features, competency questions and use case scenarios.

The ontology is freely available at: http://www.scai.fraunhofer.de/en/business-research-areas/bioinformatics/downloads.html (owl files) and http://bishop.scai.fraunhofer.de/scaiview/ (browser).

**Conclusions:**

HuPSON provides a framework for a) annotating simulation experiments, b) retrieving relevant information that are required for modelling, c) enabling interoperability of algorithmic approaches used in biomedical simulation, d) comparing simulation results and e) linking knowledge-based approaches to simulation-based approaches. It is meant to foster a more rapid uptake of semantic technologies in the modelling and simulation domain, with particular focus on the VPH domain.

## Background

Biomedical ontologies have proven their value in diverse applications as metadata annotation and data integration [1], knowledge representation [2], and knowledge discovery [3]. Ontologies also play a fundamental role in harmonizing name spaces, shared semantics and standardization of data and of model resources [4]. Recently, analysis of mechanical problems in a human body under disease conditions, using computational algorithms and models, has gained momentum in biomechanics research [5].

Many well-established ontologies exist in the biomedical domain that can be used to annotate simulation experiments on the anatomical, molecular, chemical, phenotypic levels (see, e.g., the BioPortal repository [6]). However, despite the fast growth in the number of biomechanical studies, there exist only a few semantic frameworks explicitly developed for simulation experiments and models. Examples include the Kinetic Simulation Algorithm Ontology (KiSAO) [7], the Terminology for the Description of Dynamics (TEDDY) [7], the Discrete-Event Modeling Ontology (DeMO) [8,9] and the Systems Biology Ontology (SBO) [7,10]. DeMO formalizes information only related to discrete systems, KISAO is limited in scope to kinetic models and algorithms, TEDDY deals with classification of dynamic features in simulation and SBO represents model components. There also exists the Living Human Digital Library (LHDL) domain ontology [11,12] that serves as a foundation for coherent annotation of LHDL resources and their retrieval and traceability. Subsequently, it is very specific to the LHDL project requirements.

The RICORDO interoperable anatomy and physiology project [13] provides tools that help physiology and pharmacology researchers and medical students in the semantic interoperability of clinical data and model resources. RICORDO combines concepts from standard ontologies to form “composites”, thus creating more complex concepts such as “venous return” [13]. The approach of “composite annotations” is also proposed by Gennari et al. [14]. The authors explicitly avoid constructing a biosimulation ontology, instead they leverage established ontologies to circumvent the combinatorial challenge of having to include all possible multi-term class names, such as “aortic blood pressure”. The SemSim approach [15] makes use of such composite annotations, annotating model parameters, variables and other observables against terms from reference ontologies. The aim of SemSim is to create semantic interoperability of biosimulation models by creating machine-readable definitions. While this is a valid approach to creating interoperability and the integration of resources, the problem remains that semantic information is spread among different external sources and an additional tool (e.g. SemGen [14], the RICORDO toolkit [13]) is needed.

None of the above works provides a comprehensive ontology that covers simulations and algorithmic approaches. We believe that a “stand-alone” ontology, versus semantic tools that leverage existing ontologies in a distributed way, that covers the biosimulation domain and algorithmic approaches will be a useful tool and will serve interested groups involved in cross-disciplinary simulation initiatives. An example of such an initiative is the VPH [16]. The VPH foresees that modelling and simulations will enable a better understanding of the human’s body’s functioning and its pathological processes, as well as help develop therapies and tools that can aid disease diagnosis, treatment and prevention. Thus, in order to support these types of initiatives, we developed and evaluated an initial version of the Human Physiology Simulation Ontology (HuPSON).

## Results

### Scope and purpose

HuPSON provides a framework for a) annotation of simulation experiments with standard ontology terms, b) text-mining based information retrieval that is required for modelling, c) interoperability of algorithmic approaches used in biomedical simulation, d) comparability of simulation results and interoperability on different structural scales (from the human anatomy down to cells and molecules) and e) linking knowledge-based approaches (e.g. ontologies) to simulation-based approaches (e.g. differential equation-based approaches).

The current primary use of HuPSON is to aid in text-mining (scope b)). Scopes a) and b) are validated in the Results section below, whereas for a discussion of scopes c)-e), the reader is referred to the Discussion section.

### Ontology contents

**Figure 1:**
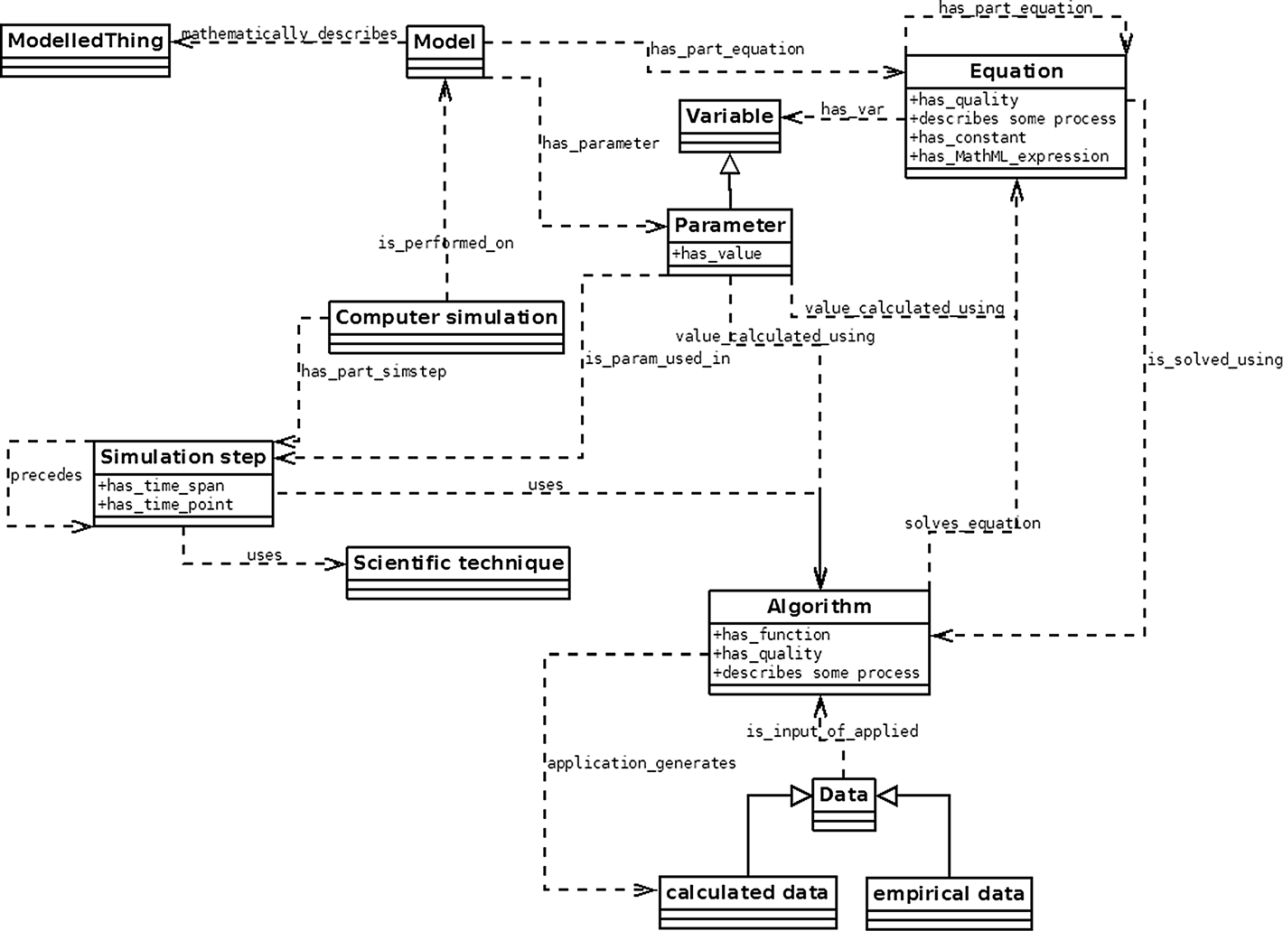
**Extract of diagram used for modelling HuPSON.** UML-like diagram used for the design of HuPSON – relationships between the upper-level classes *model*, *biomedical computer simulation*, *equation*, *parameter*, *scientific technique*, *algorithm*, and related classes; normal arrows denote subsumption relations, dotted arrows denote object properties that hold between the two classes.

The ontology was modelled using a UML-type of diagram as shown in Figure 1. A computer simulation consists of simulation steps that use algorithms and scientific techniques and is performed on a model. A model mathematically describes some modelled thing, which can be an anatomical part, a process, function, or a quality. A model has equations and parameters. A list of definitions of these main ontology classes is given in Table 1.

**Table 1:** Main ontology classes

**Ontology class**	**Definition**
Computer simulation	“A broad collection of methods used to study and analyze the behavior and performance of actual or theoretical systems. Simulation studies are performed, not on the real-world system, but on a (usually computer-based) model of the system created for the purpose of studying certain system dynamics and characteristics. […]”
Simulation step	“A specific stage of progression through a sequential process” of a simulation.
Algorithm	An algorithm is a set of instructions, sometimes called a procedure or a function, that is used to perform a certain task. […]
Scientific technique	A scientific technique is any systematic method to obtain information of a scientific nature or to obtain a desired material or product. […]
Model	A mathematical model is “the use of mathematical language to describe the behaviour of a system. A mathematical model usually describes a system by a set of variables and a set of equations that establish relationships between the variables. “ […]
Modelled thing	Thing that is mathematically described via a model.
Equation	A statement asserting the equality of two expressions, usually written as a linear array of symbols that are separated into left and right sides and joined by an equal sign.
Parameter	Any value passed to a program by the user or by another program in order to customize the program for a particular purpose. […]

Definition of main HuPSON classes.

**Figure 2:**
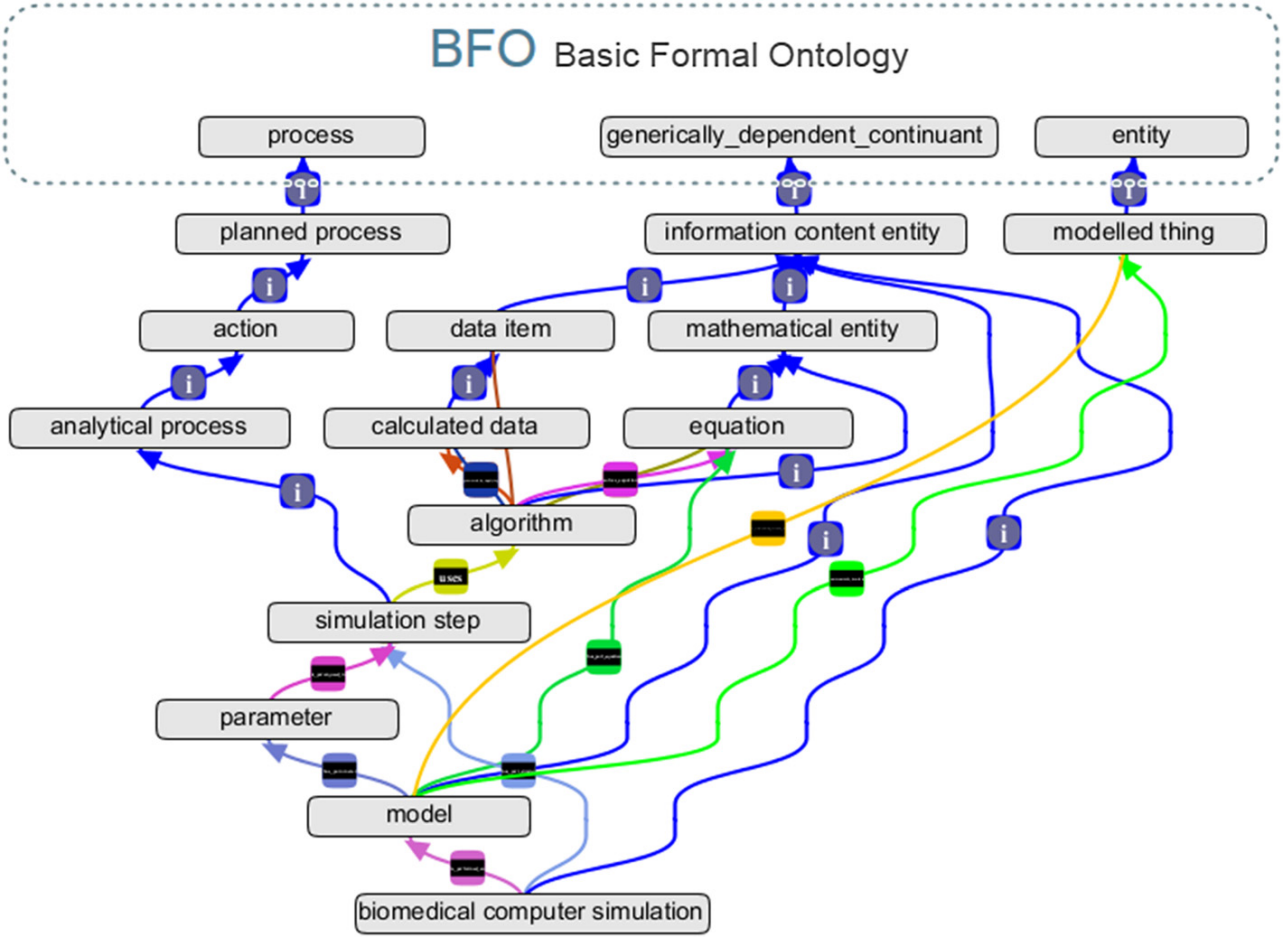
**HuPSON hierarchy plugged into BFO.** HuPSON class hierarchy depicting classes biomedical computer simulation, algorithm, equation, model and related classes inside the BFO hierarchy, displayed with OBO Graph View [18] inside Protégé; blue arrows diplaying “i”: subclass relations; light blue; has_part_simstep; light green: mathematically_describes; green: has_part_equation; yellow: uses; orange: is_mathematically_described_by; brown: application_generates; light violet: is_performed_on; violet: is_param_used_in (bottom)/solves_equation (top).

**Figure 3:**
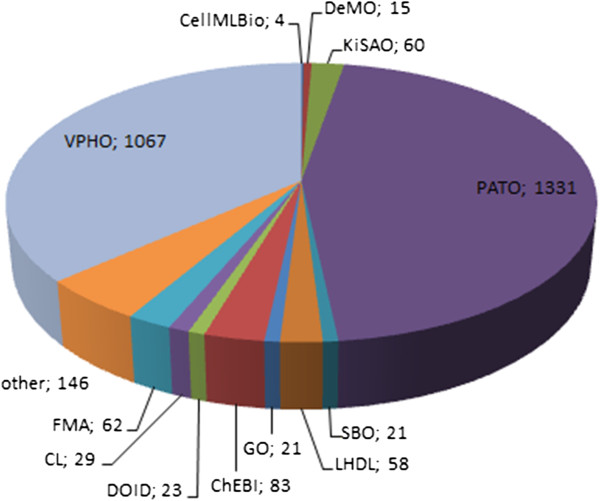
**Class provenance in HuPSON.** This diagram shows the provenance of classes. 36% of all classes were added manually inside the HuPSON namespace, the other 64% stem from related ontologies. “other” includes further ontologies/taxonomies such as the NCBI taxonomy, Ontology for Biomedical Investigations (OBI), Unit Ontology (UO) and Medical Dictionary for Regulatory Affairs (MEDDRA) (all available from BioPortal).

The ontology (cf. Figure 2) contains 2,920 classes and a total of 7,262 synonyms. 1,067 (36%) of these classes were added manually, whereas the other 64% of classes were integrated from related ontologies (Figure 3). Wherever possible, “leaf” equation classes were annotated via an annotation property with their corresponding MathML [17] expression. Approximately 55% of the 108 equations have a MathML expression associated to them. In addition to textual definitions, axioms have been inserted wherever they are deemed meaningful (both necessary and sufficient axioms and class-descriptive axioms). For instance, the class ‘computational fluid dynamics (CFD) model’ is described via has_part_equation some ‘numerical equation’ and mathematically_describes some ‘hydrodynamic quality’, allowing the reasoner to infer that it is both a ‘hydrodynamic model’ and a ‘numerical model’, as those classes are defined via according necessary and sufficient axioms.

### Validation

The HermiT reasoner [19] was used to ensure ontology consistency. The ontology was evaluated based on structural features^a^ and with regard to its performance on text-mining tasks. Relatively high values of class number (2,920), leaves (1,927), maximum width (727) and average width (270.05), along with a fanout factor of 0.71, are indicative of the ontology's broad coverage; similarly, the depth values of 10 (max.) and 5.5 (avg.) are indicators of a relatively good specificity of types to the domain.

The screenshot provided as Additional file 1 is an example of a PubMed abstract annotation using HuPSON terms, and is an example of how HuPSON can be used in regard to scope a). Such annotations, applied to real simulation settings, also pave the grounds for comparability of simulation experiments by leveraging the semantics from the ontology (scope d)).

As an example of HuPSON’s applicability to relevant text-mining tasks (scope b)), 700 PubMed abstracts about simulations in the VPH context were downloaded from MEDLINE [20] and used to produce our own gold standard (i.e. training and test sets) for evaluation. This gold standard consists of the set of annotations that are expected when running a text-mining tool that queries for the HuPSON terms over the abstracts. Calculation of the system performance resulted in a recall, a precision and an F-score of around 0.66 in the test set. Furthermore, participants from different working groups, whom participated in the VPH Network of Excellence, were asked to provide queries typical for the VPH domain (see competency questions/queries in Table 2). To study these real-use case scenarios, ProMiner [21], using the HuPSON dictionary (see Methods section) as input, was applied to the complete MEDLINE abstracts for the identification of specific knowledge. The recognized concepts from the HuPSON dictionary were visualized using SCAIView semantic search engine [22]. Table 3 shows that both ontology-based queries resulted in more true positive hits than their PubMed counterparts. These abstracts are considered to represent an “information gain” compared to the PubMed query results. Moreover, HuPSON was used in SCAIView to retrieve studies that report on heart biomechanics modelling, with a specific focus on the application of mechanical pump models to supporting blood circulation in human hearts. Starting with the query [“heart” AND “pump model” AND “blood circulation”], the retrieved studies were further filtered for “Homo sapiens”, resulting in 9 identified documents that correctly describe blood pump models and their application to blood circulation in human hearts (i.e. PMIDs: 10203406, 18002874, 7872572, 17938774, 17015490,15802261, 2752563, 18401072, and 11940364). The retrieved information can help experts improve their understanding of the applicability of such models and the underlying mechanical theory (for examples, see findings in [23] (PMID: 18002874) and [24] (PMID: 11940364), Additional file 2). Note that using an ontology-driven semantic system to search the knowledge space of publications, using complex queries, outperforms traditional search engines such as that offered by the PubMed system in targeted information retrieval. Exemplifying this is that PubMed, using the same search query as described above, finds only one abstract (i.e. PMID: 10203406).

**Table 2:** Competency questions

**Query for competency question expressed in free text**	**HuPSON-based query**	**Query in PubMed**
Search the literature for fluid structure interaction models of the aneurysm simulating the pressure and its link to rupture	((fluid–structure interaction (FSI) model) AND pressure AND ruptured AND aneurysm)	(“fluid–structure interaction model” OR “fluid structure interaction model”) and aneurysm and pressure and ruptured
Find publications on velocity of blood flow and rupture outcomes of aneurysms	(velocity AND (ruptured OR unruptured) AND aneurysm AND (blood circulation))	velocity AND (ruptured OR unruptured) AND aneurysm AND “blood circulation”

Selected competency questions formulated by VPH experts and transformed into HuPSON-based queries and PubMed queries.

**Table 3:** Evaluation via competency questions

**Query expressed in free text**	**Hits of SCAIView query**	**Hits of PubMed query**
Search the literature for fluid structure interaction models of the aneurysm simulating the pressure and its link to rupture	8/9TP* ^a^	0/0 TP*
Find publications on velocity of blood flow and rupture outcomes of aneurysms	29/59 TP* ^b^	2/3 TP*

Competency questions evaluated in SCAIView based on HuPSON, and PubMed queries; *TP meaning ”informative“ and “relevant“ to the query.

^a^corresponding PMIDs: 16712729, 18568827, 16221475, 16121537, 16500664, 16153654, 21722905, 21088917.

^b^corresponding PMIDs: 9647316, 19563706, 21096182, 1644550, 19675980, 19329152, 16783935, 18350286, 16813443, 17047283, 21233477, 10447563, 10414574, 18787954, 19553143, 12695182, 21071533, 20508183, 21161794, 17416810, 17885239, 18977588, 18622621, 10472991, 16321205, 20435277, 19762460, 20300847, 19936925.

Lastly, in order to show the applicability of HuPSON to independent domains, we applied it to Alzheimer’s disease by challenging the system to retrieve and semantically filter the published knowledge related to simulation and modelling within this domain. Alzheimer’s disease is a common neurological disorder afflicting the elderly, whose clinical diagnosis is problematic because of overlapping early symptoms with other diseases. However, structural imaging has been recently shown to be a valuable tool in differential diagnosis of most dementias [25]. To identify studies reporting the application of image analysis models to the differential diagnosis of Alzheimer’s using MRI, we used the MeSH terminology in conjunction with HuPSON and performed a query in the SCAIView environment. 18 of the 23 retrieved abstracts were relevant to the query and correctly identified such studies. From these documents, we were able to extract what specific model types are used in the query context (e.g. “network diffusion models” and “logistic regression models”). This kind of information can help model developers choose an appropriate model for their research.

## Discussion

HuPSON provides ontology classes that describe things that can be modelled. These include a human’s anatomical parts, from gross anatomy down to the molecular level, physiological processes, functions and qualities. It brings together, into one comprehensive ontology, external ontologies and adds new classes that are not available elsewhere, but are important for simulations. Classes have been chosen in a methodological way from relevant literature and complemented by terms considered important by representatives of the VPH community. Such selection helps to ensure that the terms contained in the ontology reflect the way that they are commonly expressed and used by the community. Moreover, it ensures that those composites that are most commonly mentioned in the literature are contained in the ontology. The approach of converting the ontology classes and their synonyms into a dictionary file make the ontology ready for use in text mining approaches. Re-use of external ontology class URIs makes it interoperable with external established ontologies. The hierarchical mathematical model types are associated to the equation types that are solved inside them, the equations, in turn, are associated to their MathML descriptions (approach similar to that described by Ivchenko et al. [26]). The equations are thus computer-readable and are, furthermore, placed in their correct hierarchical context. This makes them available to semantically-aware computer processing. In doing so, we propose a solution to connect the semantics and knowledge-driven approaches to the simulation approaches that typically employ differential equations (scopes c)-e)).

One reason for relatively low values of precision and recall in its evaluation lies in the simulation domain’s broadness and the complexity of the terms used therein; a term such as “mechanical, trileaflet heart valve prosthesis”, even though specific to the domain, does not appear in many scientific simulation-related texts and thus, is not present among the synonyms.

## Conclusions

HuPSON is meant to foster a more rapid uptake of semantic technologies in the modelling and simulation domain in general, with a particular focus in the VPH domain. The ontology is suited to link the mathematics and algorithmics behind biomedical simulations and the communication dealing with simulation experiments. It can be used to systematically detect various types of statements in scientific reports and publications. One future application of the ontology could be the systematic detection of assumptions made in modelling and simulations. This is quite challenging since most assumptions are implicitly made. The importance of making assumptions explicit in biosimulation models was recently discussed in context to the formulation of a model’s semantics (the authors call this “meaning facets”) [27]. In HuPSON terms, for instance, one might detect the modelling assumption of Newtonian blood viscosity that is made for a model that mathematically_describes some ‘blood circulation’ and has_part some ‘Newtonian fluid dynamic equation’ (from the latter the reasoner automatically infers it to be a ‘Newtonian model’).

Finally, the perspective of “reasoning over algorithmic approaches”, based on HuPSON’s hierarchy of equations that are directly accessible to computer processing via MathML, is quite fascinating. We invite the modelling and simulation community to provide use cases to enable us to explore this possibility further. For instance, an interesting feature will be to improve the semantic enrichment of equations and to connect them with more detail to variable or constant types or instances.

Note that HuPSON is meant to be a draft ontology that is proposed to the modelling and simulation community. Ontologies represent a certain view on a topic and a certain state of knowledge within a domain. The authors explicitly express that their view on the simulation domain is not the only one. Moreover, the authors are aware of the fact that new knowledge, including new algorithmic approaches, is constantly added to the biomedical simulation area. Therefore, we encourage the community to actively take up and optimize this first version of the ontology (via the BioPortal project web site), including its evaluation in real use case scenarios.

## Methods

### Use of tools and reasoning

To construct the OWL ontology, Protégé 4.1.9 (Build 209) [28] together with its inbuilt HermiT 1.3.3 reasoner were used. For evaluation purposes, ProMiner was used as a named entity recognition (NER) tool and SCAIView as a literature mining environment that allows for a context-sensitive document retrieval based on ontologies.

Although there does not exist any single standard for the evaluation of ontologies (cf., NCBO Ontology Summit 2013 [29] on ontology evaluation), there are various proposals for how an ontology might be evaluated (e.g., [29,30], and [31], or the discussion by Hoehndorf et al. [32]). In [31], the authors state that “good ontologies are the ones that serve their purpose” and in [32] it is stated that evaluation of (‘applied’) ontology will “depend on the desired application”. As the current primary purpose of HuPSON is to aid in text-mining, its evaluation was focused mainly on how it performed with regard to literature-based mining of simulation knowledge. This was accomplished using competency questions formulated in advance by VPH experts and by use cases. For gold standard creation (i.e. a training set and a test set), 700 PubMed abstracts about simulations in the VPH context were downloaded from MEDLINE. The ontology class labels and synonyms were converted into a dictionary format, then these terms were searched in both training set and test set using ProMiner. The NER search was performed using case-insensitive, word order-sensitive and longest string exact match search constraints. For calculation of precision, recall and F-score of the test set, the following formulas were used:

$$\mathrm{Precisio}n^{f}=TP^{c}/\left(\mathrm{TP}+FP^{d}\right)$$

$$\mathrm{Recal}l^{g}=\mathrm{TP}/\left(\mathrm{TP}+FN^{e}\right)$$

$$F‒\mathrm{scor}e^{h}=\left(2*\mathrm{Precision}*\mathrm{Recall}\right)/\left(\mathrm{Precision}+\mathrm{Recall}\right).$$

The MathML code contained within the ontology was generated from equations collected from the literature and encoded with the help of SnuggleTeX 1.2.2 [33]. SnuggleTeX is an open-source java library that converts LaTeX into semantically enriched MathML, or ContentMathML wherever the conversion can be done automatically. Equations that have been annotated with MathML code via an annotation property also have a textual definition and are annotated with a PubMed ID pointing to relevant literature.

Ranking of n-grams was performed using the Porter Stemmer [34]. Noun phrase chunking was done using a chunker based on the OpenNLP system [35].

The reasoner was used to subsume types with class-descriptive axioms to be a subtype of formally defined ones via necessary and sufficient axioms. In other words, (secondary) classification is left to the reasoner and ontology maintenance is eased through avoidance of direct multiple inheritance assertions, as proposed as a good practice for modularised ontology construction [36]. Axioms necessary for this purpose were added manually, for instance, to classes with composite multi-term labels.

### Knowledge acquisition and conceptualization

In order to identify relevant entities and to ensure that HuPSON will cover the most important terms from existing related work, standards for simulation and modelling (such as SED-ML, Cell-ML, SBML, MIASE, MIRIAM, cf. [16]), domain ontologies [6] in the field (cf. External ontologies section) and relevant literature were studied. A corpus of pertinent literature articles and publications in the context of the official VPH Network of Excellence and other VPH projects was collected and analysed manually for candidate upper-level classes. Around 32,000 relevant PubMed abstracts were queried for candidate subclasses of these upper-level classes (bigram to 5-gram word combinations containing the top-level class terms as the last word of the n-gram, using a Java program written for this purpose). Found n-grams were sorted by occurrence and subsequently ranked. To ensure the ontology covers the most important entities in the simulation context, approximately 15,000 of the abstracts from various resources including the ones used in the n-gram search, VPH project websites (e.g., VPH NoE, Biomed Town, LDL) and extra information disseminated through existing VPH projects (e.g., RICORDO, euHeart, VPHOP, ARTreat, preDiCT and others^b^) were analysed using a noun phrase chunker. Thus, composite terms that are often used in the literature, and subsequently important for text mining, found their way into the ontology. For synonym enrichment of ontology classes, an approach was chosen that combines manual synonym annotations with the use of external annotation services offered by the National Center for Biomedical Ontology (NCBO) [37].

### External ontologies

URIs of external ontologies have been re-used, where appropriate, according to the Minimum Information to Reference an External Ontology Term (MIREOT) principles [38] (cf. Figure 3). These include: CellMLBio Ontology [39], DeMO [8,9], KiSAO [7], the Phenotypic Quality Ontology (PATO) [40], Systems Biology Ontology (SBO) [7] and LHDL Master Ontology [11,12]; Gene Ontology (GO) [41], Chemical Entities of Biological Interest (ChEBI) [42], Human disease ontology (DOID) [43], Cell type ontology (CL) [44] and the Foundational Model of Anatomy (FMA) [45]. For model types, algorithm types and qualities, the entire DeMO, KiSAO and PATO hierarchical structures were included in HuPSON. Further information on included external ontology classes is provided separately (Additional file 3).

The Basic Formal Ontology (BFO) [46] was preferred over other upper-level ontologies (e.g. DOLCE [47], SUMO [48], the General Formal Ontology [49] and Cyc [50]) because of its use within the OBO community that follows the OBO principles [51], its large user base and the many ontologies that meanwhile have been constructed on BFO under the OBO Foundry [51] umbrella. Using BFO upper levels, interoperability to those resources is ensured. Relations were also adopted from established standards, such as rdf-schema [52], Dublin Core (DC) [53] and the OBO Foundry Relation Ontology (RO) [54], as far as possible.

## Endnotes

^a^number classes (without owl:Thing): 2920; number roots: 10; number leaves: 1927; max width/breadth: 727; avg. width/breadth: 270.05; max depth: 10; total no. children: 2885; avg. number children: 1.068; avg. depth (avg. root-to-leaf distance): 5.486; depth variance (var(d) = E[d^2]-E[d]^2): 2.637; width/breadth variance (var(w) = E[w^2]- E[w]^2): 55455850; tangledness (no. nodes with 2+ parents/total no. nodes): 0.060; fanout factor (no. leaf classes/number classes): 0.713.

^b^for a complete list see http://www.vph-noe.eu/vph-projects.

^c^number of true positive hits correctly found, i.e., matching the annotation in the gold standard.

^d^number of false positive hits, i.e., hits found but not contained in the gold standard.

^e^number of false negative hits, i.e., entities not found but contained in the gold standard.

^f^proportion of correct hits out of all hits.

^g^proportion of correct hits out of all terms that should have been correctly found.

^h^overall measure of accuracy (harmonic mean of precision and recall).

## Competing interests

The authors declare that they have no competing interests. This work was not funded by the EU VPH programme.

## Authors’ contributions

MG designed and coded the ontology, contributed to its evaluation and drafted the manuscript. EY and AM contributed to evaluation and to manuscript drafting. JW, HL and BZ carried out text annotations. BdB contributed to ontology design. HTM performed text mining. MHA participated in the design of the study and revised the paper critically. All authors read and approved the final manuscript.

## Supplementary Material

Additional file 1 Abstract of a simulation publication regarding wall sheer stress in aortic coarctation patients annotated with HuPSON terms, displayed in SCAIView environment. Click here for file

Additional file 2 **HuPSON-driven information retrieval scenario for the application of mechanical pump models to supporting blood circulation in human hearts, displayed in SCAIView environment.** The screenshot shows an exemplary document retrieved by the following HuPSON-driven query: [“heart” AND “pump model” AND “blood circulation”]. HuPSON classes found in the PubMed abstract are highlighted in green. Click here for file

Additional file 3 External ontologies. Click here for file
