# Supplementary material for: HuPSON: the human physiology simulation ontology
Source: J Biomed Semantics. 2013 Nov 22;4:35. doi: 10.1186/2041-1480-4-35 (PMC4177144; doi:10.1186/2041-1480-4-35)
Supplement: Additional file 1 — Abstract of a simulation publication regarding wall sheer stress in aortic coarctation patients annotated with HuPSON terms, displayed in SCAIView environment. [file 2041-1480-4-35-S1.pdf]

1 . Computational simulations demonstrate altered wall shear stress in aortic coarctation patients treated by resection with end-to-end anastomosis.

PubMed PubMedCentral 21801315 **Authors:** LaDisa, John F; Dholakia, Ronak J; Figueroa, C Alberto; Vignon-Clementel, Irene E; Chan, Frandics P; Samyn, Margaret M; Cava, Joseph R; Taylor, Charles A; Feinstein, Jeffrey A **Date:** -- **Journal:** Congenital heart disease **Affiliation:** Department of Biomedical Engineering, Marquette University, Children's Hospital and the Medical College of Wisconsin, 1515 West Wisconsin Avenue, Milwaukee, WI 53233, USA. john.ladisa@marquette.edu

☐ Statistics

BACKGROUND: Atherosclerotic plaque in the descending thoracic aorta (dAo) is related to altered wall shear stress (WSS) for normal patients. Resection with end-to-end anastomosis (RWEA) is the gold standard for coarctation of the aorta (CoA) repair, but may lead to altered WSS indices that contribute to morbidity. METHODS: Computational fluid dynamics (CFD) models were created from imaging and blood pressure data for control subjects and age- and gender-matched CoA patients treated by RWEA (four males, two females, 15 ± 8 years). CFD analysis incorporated downstream vascular resistance and compliance to generate blood flow velocity, time-averaged WSS (TAWSS), and oscillatory shear index (OSI) results. These indices were quantified longitudinally and circumferentially in the dAo, and several visualization methods were used to highlight regions of potential hemodynamic susceptibility. RESULTS: The total dAo area exposed to subnormal TAWSS and OSI was similar between groups, but several statistically significant local differences were revealed. Control subjects experienced left-handed rotating patterns of TAWSS and OSI down the dAo. TAWSS was elevated in CoA patients near the site of residual narrowings and OSI was elevated distally, particularly along the left dAo wall. Differences in WSS indices between groups were negligible more than 5 dAo diameters distal to the aortic arch. CONCLUSIONS: Localized differences in WSS indices within the dAo of CoA patients treated by RWEA suggest that plaque may form in unique locations influenced by the surgical repair. These regions can be visualized in familiar and intuitive ways allowing clinicians to track their contribution to morbidity in longitudinal studies.
