# Supplementary material for: HuPSON: the human physiology simulation ontology
Source: J Biomed Semantics. 2013 Nov 22;4:35. doi: 10.1186/2041-1480-4-35 (PMC4177144; doi:10.1186/2041-1480-4-35)
Supplement: Additional file 2 — HuPSON-driven information retrieval scenario for the application of mechanical pump models to supporting blood circulation in human hearts, displayed in SCAIView environment. The screenshot shows an exemplary document retrieved by the following HuPSON-driven query: [“heart” AND “pump model” AND “blood circulation”]. HuPSON classes found in the PubMed abstract are highlighted in green. [file 2041-1480-4-35-S2.pdf]

3 . Pump models assessed by transesophageal echocardiography during cardiopulmonary resuscitation.

PubMed PubMedCentral 11940364 **Authors:** Liu, Pinming; Gao, Yan; Fu, Xiangyang; Lu, Junhao; Zhou, Ying; Wei, Xianglong; Li, Gongxin; Ding, Mingxue; Wu, Hongchao; Ye, Wensheng; Liu, Yingfeng; Li, Zhiliang **Date:** 2002-03 **Journal:** Chinese medical journal **SciMago: Affiliation:** Department of Cardiology, Zhongshan University, Guangzhou 510120, China. liupinming@hotmail.com

☐ Statistics

OBJECTIVE: Transesophageal echocardiography was performed during closed-chest cardiopulmonary resuscitation (CPR) in in-hospital cardiac arrest to further explore the hemodynamic mechanism of CPR.

METHODS: CPR attempts were performed according to advanced cardiovascular life support guidelines in 6 cases of in-hospital cardiac arrest. Multi-plane transesophageal echocardiography was carried out within 15 min of initiation of CPR. Throughout CPR, the motion of the mitral, tricuspid and aortic valves, the changes in the left ventricular cavity size and the thoracic aortic diameter were observed. Trans-mitral and trans-aortic Doppler files of blood flow were also documented.

RESULTS: A closure of the mitral and tricuspid valves with simultaneous opening of the aortic valve occurred exclusively during chest compression, resulting in forward blood flow in the pulmonary and systemic circulation. Peak forward aortic flow at a velocity of 58.8 +/- 11.6 cm/s was recorded during the compression phase. Whereas, a closure of the aortic valve and rapid opening of the atrioventricular valves associated with ventricular filling during relaxation of chest compression was noted in all 6 patients. Peak forward mitral flow at a velocity of 60.6 +/- 20.0 cm/s was recorded during the release phase. Mitral regurgitation during the chest compression period was detected in 5 patients, reflecting a positive ventricular-to-atrial pressure gradient. A reduction in the left ventricular chamber and an increase in the thoracic aortic diameter during the compression phase was found in all patients, indicating that direct cardiac compression contributed to forward blood flow.

CONCLUSION: These observations favor the cardiac pump theory as the predominant hemodynamic mechanism of forward blood flow during CPR in human beings.
